# Down-Regulating HAUS6 Suppresses Cell Proliferation by Activating the p53/p21 Pathway in Colorectal Cancer

**DOI:** 10.3389/fcell.2021.772077

**Published:** 2022-01-12

**Authors:** Aling Shen, Liya Liu, Yue Huang, Zhiqing Shen, Meizhu Wu, Xiaoping Chen, Xiangyan Wu, Xiaoying Lin, Youqin Chen, Li Li, Ying Cheng, Jianfeng Chu, Thomas J. Sferra, Lihui Wei, Qunchuan Zhuang, Jun Peng

**Affiliations:** ^1^ Academy of Integrative Medicine, Fuzhou, China; ^2^ Fujian Key Laboratory of Integrative Medicine in Geriatrics, Fujian University of Traditional Chinese Medicine, Fuzhou, China; ^3^ Department of Pediatrics, Case Western Reserve University School of Medicine, Rainbow Babies and Children’s Hospital, Cleveland, OH, United States; ^4^ Department of Health Management, Fujian Provincial Hospital, Fuzhou, China

**Keywords:** HAUS6, tumor growth, proliferation, p53/p21 pathway, survival

## Abstract

**Background:** HAUS6 participates in microtubule-dependent microtubule amplification, but its role in malignancies including colorectal cancer (CRC) has not been explored. We therefore assessed the potential oncogenic activities of HAUS6 in CRC.

**Results:** HAUS6 mRNA and protein expression is higher in CRC tissues, and high HAUS6 expression is correlated with shorter overall survival in CRC patients. HAUS6 knockdown in CRC cell lines suppressed cell growth *in vitro* and *in vivo* by inhibiting cell viability, survival and arresting cell cycle progression at G0/G1, while HAUS6 over-expression increased cell viability. We showed that these effects are dependent on activation of the p53/p21 signalling pathway by reducing p53 and p21 degradation. Moreover, combination of HAUS6 knockdown and 5-FU treatment further enhanced the suppression of cell proliferation of CRC cells by increasing activation of the p53/p21 pathway.

**Conclusion:** Our study highlights a potential oncogenic role for HAUS6 in CRC. Targeting HAUS6 may be a promising novel prognostic marker and chemotherapeutic target for treating CRC patients.

## Background

Colorectal cancer (CRC) is one of the leading causes of mortality worldwide (Chouinard et al., 2013). Progression from microscopic pre-neoplastic lesions to larger adenomas involves a cascade of mechanisms including tumor suppressor gene mutation and bypass of cell cycle checkpoints (Fakih, 2015). Unfortunately, current treatment strategies have low success rates due to our poor understanding of the molecular mechanisms behind CRC progression. Hence, there is an urgent demand to understand the fundamental mechanisms underlying CRC development and to find novel molecular targets (Vescovo and MA, 2015).

Follow-up analysis of gene expression profile microarrays from our previous study (GEO Submission: GSE113513) (Shen et al., 2019) showed that levels of mRNA encoding HAUS augmin like complex subunit 6 (HAUS6) are higher in CRC tissues than in adjacent noncancerous colorectal tissues. Our preliminary experiments further suggested that HAUS6 knockdown inhibits growth of CRC cells in culture (data not shown). These results suggest that HAUS6 has oncogenic potential in CRC.

HAUS6 is one of the 8 subunits of the augmin complex (Goshima et al., 2008; Zhu et al., 2008), which is required for microtubule (MT)-dependent MT amplification during cell division independently of the centrosomes and chromatin (Lawo et al., 2009; Du et al., 2011; Nakaoka et al., 2012; Hayward et al., 2014). HAUS6 promotes MT-dependent MT amplification by interacting with and recruiting neural precursor cell-expressed developmentally down-regulated protein 1 (NEDD1), a subunit of the NEDD1-γ-tubulin ring complex. Depletion of HAUS6 reduces MT density in the bipolar spindle (Zhu et al., 2008). Dissociation of the augmin complex or the presence of HAUS6 and/or γ-tubulin reduces MT formation and MT bundling, thereby affecting spindle assembly during mitosis and MT organization in interphase (Goshima and Scholey, 2010; Sá nchez-Huertas et al., 2016). MTs, spindle poles and chromosomes form the main elements of the spindle architecture (Kline-Smith and Walczak, 2004; Meunier and Vernos, 2012), which plays an essential role in organizing bipolar spindles and chromosomal segregation during mitosis (Nakayama et al., 2013). Failure of these processes results in mitotic arrest and apoptosis and contributes to chromosome mis-segregation and aneuploidy, a hallmark of cancer (Holland and Cleveland, 2012). However, the role of HAUS6 in cancer has never been evaluated to our knowledge. In the present study, we investigated the effects of HAUS6 expression on patient prognosis and on viability and proliferation of CRC cell lines *in vitro* and *in vivo*, as well as its potential underlying mechanisms.

## Materials and Methods

### Bioinformatic Analysis

HAUS6 expression in CRC and noncancerous colorectal tissues was compared using the Oncomine database (www.oncomine.org), as described previously (Shen et al., 2019) (Supplementary Table S1). Correlation of HAUS6 expression with CRC patient survival was performed using the R2 Bioinformatic Platform (http://r2.amc.nl), as described previously (Shen et al., 2019). The threshold of significance was set at *p* < 0.05.

### Patients and Specimens

Both CRC and matched adjacent non-cancerous tissues were collected from 36 CRC patients admitted to the First People’s Hospital Affiliated with Fujian University of Traditional Chinese Medicine (FJTCM). Clinicopathologic characteristics of patients are summarized in Supplementary Table S2. No patients received radio- or chemotherapy prior to surgery. Tissues were snap-frozen and stored in liquid nitrogen for quantitative RT-PCR or processed for immunohistochemistry. Samples were stained with hematoxylin and eosin, and then examined by experienced pathologists, who assigned pathologic tumor-node-metastasis (p-TNM) grades according to International Union Against Cancer guidelines.

In addition, cDNA from 80 primary CRC and 15 adjacent noncancerous colorectal tissues were obtained from the cDNA-HColA095Su01 tissue cDNA array (Shanghai Outdo Biotech, Shanghai, China; Supplementary Table S3). A further 280 primary CRC and 260 adjacent noncancerous colorectal tissues were obtained from the tissue microarray (TMA) (Shanghai Outdo Biotech; Supplementary Table S4).

### Quantitative RT-PCR

Cells and tissue samples were lysed with RNAiso Plus reagent (Takara, Beijing, China) and total RNA was extracted according to the manufacturer’s instructions. A total of 1 μg RNA was reverse-transcribed into cDNA using PrimeScript RT reagent kit (Takara, Beijing) and RT-qPCR was performed using SYBR Premix Ex Taq (Takara, Beijing) and the ABI 7500 Fast Real-Time PCR System (Applied Biosystems, Carlsbad, CA, United States) according to manufacturer protocols. Primer sequences against HAUS6, TP53, CDKN1A and GAPDH are shown in Supplementary Table S5. GAPDH was used as an internal control.

### Immunohistochemical Staining

Tissues were processed using routine immunohistochemistry methods as in our previous study (Shen et al., 2019). Tissue specimens collected in our laboratory were fixed, sectioned and stained with primary antibodies against HAUS6 and Ki-67 then secondary biotinylated antibody (Supplementary Table S6), followed by incubation with horseradish peroxidase-conjugated streptavidin (Maixing, Fuzhou, China). Background staining was assessed by omitting the primary antibody. Five randomly selected fields were examined using a Leica microscope (Wetzlar, Germany) at ×40 magnification.

For tissue arrays, slides were incubated with primary antibody against HAUS6 (dilution 1:500, Abcam, United Kingdom) as described (Wang et al., 2014; Shen et al., 2019). Images were captured using a Nano Zoomer 2.0 HT slide scanner (Hamamatsu Photonics, Hamamatsu, Japan) and processed using Nano Zoomer Digital Pathology View 1.6 software. Staining intensity and the percentage of positively stained cells were determined as described (Huang et al., 2016; Shen et al., 2019). Protein expression was calculated by multiplying the intensity and percentage scores together, giving expression scores ranging from 0 to 12.

### Survival Analysis

The median HAUS6 expression in CRC tissues was used as the cutoff to divide tumors into “low expression” or “high expression”. The relationship between HAUS6 expression and overall survival was analyzed using Kaplan-Meier curves and the log-rank test.

### Cell Culture

Human CRC cell lines HCT116, HCT-8, HT-29, Caco2 and RKO were purchased from the Cell Bank of the Chinese Academy of Sciences (Shanghai, China). Human colon cell line FHC (ATCC No. CRL-1831) was purchased from the American Type Culture Collection (Maryland, United States). Wild-type HCT116 cells (HCT116/p53^+/+^) and p53 knockout HCT116 cells (HCT116/p53^−/−^) were a gift from Dr. Yao Lin (Fujian Normal University, Fujian, China) and originally obtained from Dr Bert Vogelstein (Johns Hopkins University, Baltimore, MD). All human cell lines have been authenticated using STR (or SNP) profiling within the last 3 years has been included. RKO, HCT116, HCT-8, Wild-type HCT116 cells (HCT116/p53^+/+^) and p53 knockout HCT116 cells (HCT116/p53^−/−^) cells were maintained in RPMI1640 (Thermo Fisher Scientific); HT-29 cell in M5’A medium (KeyGEN); Caco2 cells in DMEM (Thermo Fisher Scientific); and FHC cells in DMEM supplemented with 10 mM HEPES (for a final conc. of 25 mM), 10 ng/ml cholera toxin, 0.005 mg/ml insulin, 0.005 mg/ml transferring and 100 ng/ml hydrocortisone. All media contained 10% FBS (Thermo Fisher Scientific), 100 U/ml penicillin, and 100 mg/ml streptomycin (Hyclone) at 37°C in a humidified atmosphere of 5% CO_2_. Cells were verified using short tandem repeat genotyping and examined for *mycoplasma* contamination using RT-PCR.

5-FU (Sigma-Aldrich) was dissolved in DMSO and final concentration of 2.5 μM used to treat control and HAUS6 knockdown cells for 24h; DMSO was used as a vehicle control.

### Lentiviral Transduction

Lentiviruses encoding shRNA against HAUS6 or CDKN1A or encoding control shRNA (Supplementary Table S7 for sequences) and enhanced green fluorescent protein (EGFP) under control of the CMV promoter were obtained from Shanghai GeneChem (Shanghai, China). CRC cells were transduced with lentivirus at a multiplicity of infection (MOI) of 10 for 72 h before analysis. To examine effects of HAUS6 over-expression on cell proliferation, HT-29 cells were transduced for 72 h with a lentiviral vector (MOI = 10) encoding full-length human HAUS6 (2,946 nt, GenBank accession NM_017645; Shanghai GeneChem). Transductions were selected for 2 weeks using 1 μg/ml puromycin.

### Cell Viability Analysis

Cell viability was measured every day for 5 days by CCK-8 assay. Briefly, transduced cells were reseeded into 96-well plates at a density of 2,000 cells/well with 100 μl medium/well. CCK-8 dye (10 μl, Cell Counting Kit-8, Abbkine, Wuhan, Hebei, China) was added to each well and cells were incubated for 2 h at 37°C. Cell viability was measured at 450 nm.

### Cell Survival Analysis

Transduced cells were reseeded into 12-well or 6 well plates at a density of 500 or 1,000 cells/well and incubated for 10–12 days at 37°C under 5% CO_2_ in a humidified incubator. Colonies were fixed with 4% paraformaldehyde for 20 min and stained with 0.1% of crystal violet for 20 min. Colonies were photographed and the number of colonies counted. Data were normalized relative to control cells.

### Cell Cycle Analysis

Transduced cells were collected and fixed with cold 70% ethanol at 4°C overnight. The cells were then washed three times with PBS and incubated with FxCycle PI/RNase Staining Solution (Thermo Fisher Scientific) for 30 min. Fluorescence-activated cell sorting was performed using fluorescence activated cell sorting (FACS) Caliber (Becton Dickinson, San Jose, CA, United States), and ModfitLT version 3.0 (Verity Software House, Topsham, ME, United States) was used to analyze the cell cycle based on DNA content in different phases.

### *In Vivo* Experiments

Male pathogen-free athymic nude mice (6–8 weeks old) were purchased from Shanghai Laboratory Animal Co., Ltd. (Shanghai, China) and maintained in a specific pathogen-free (SPF) facility.

Tranduced CRC cells (1 × 10^6^) in 100 µl RPMI-1640 medium containing 50% matrigel were subcutaneously injected into the flank of nude mice (*n* = 8). Tumor growth was monitored from day 7 after injection on every second day for 19 days. Tumor volume was calculated as (1/2) (length × width^2^), where length and width refer to the longest longitudinal and transverse diameters.

Tumor growth was also measured in terms of GFP intensity using an IVIS Spectrum whole live-animal imaging system (PerkinElmer, Waltham, MA, United States). Mice were anesthetized with isoflurane and sacrificed by neck dislocation on day 26 after injection, and tumors were dissected and weighed. Collected tissues were processed for immunohistochemistry.

### Microarray Analysis

HCT116 cells were transduced with lentivirus encoding shRNA specific for HAUS6 or control shRNA for 72 h. Briefly, total RNA was extracted with Trizol reagent (Thermo Fisher Scientific) and analyzed by CapitalBio (Beijing, China) using the human GeneChip Primeview array (Affymetrix, Santa Clara, CA, United States) according to the manufacturer’s instructions. Microarray images were scanned using GeneChip Scanner 3,000 and analyzed using GeneChip GCOS 1.4 software (Affymetrix). Genes with a *p*-value < 0.05 and fold change >2 were defined as differentially expressed. The raw microarray data can be found in the NCBI Gene Expression Omnibus (GEO: GSE140326). Enriched pathways in DEGs were identified using KEGG pathway enrichment analysis.

### Western Blot Analysis

Cells were lysed with RIPA lysis buffer (Thermo Fisher Scientific) and the protein concentration was determined using BCA assay (Thermo Fisher Scientific). The proteins (50 µg) were then separated by SDS-PAGE and transferred onto a nitrocellulose membrane. The membranes were blocked with BSA (Sigma Aldrich) and incubated overnight at 4°C with primary antibody against HAUS6, p53, or p21 (Supplementary Table S6). The membranes were then washed three times with TBST (5 min per wash), and incubated at room temperature for 1 h with horseradish peroxidase-conjugated goat anti-rabbit (CST, United States). The blots were visualized using enhanced chemiluminescence (Thermo Fisher Scientific) according to the manufacturer’s protocols. Band intensities were quantified using ImageLab software. The expression of GAPDH was used as a control. Three independent experiments were performed for each assay.

### Statistical Analysis

Statistical analysis was performed using SPSS 20.0 (IBM, Chicago, IL, United States). Data are presented as mean ± SD. Differences between two groups were assessed using the independent or paired Student’s *t* test, while differences among three or more groups were assessed using one-way ANOVA. Survival differences were assessed using the log-rank test. The correlation between HAUS6 and CDKN1A expression was analyzed using Pearson’s correlation. A *p-*value of <0.05 was considered statistically significant.

## Results

### HAUS6 is Highly Expressed in CRC Tissues

**FIGURE 1:**
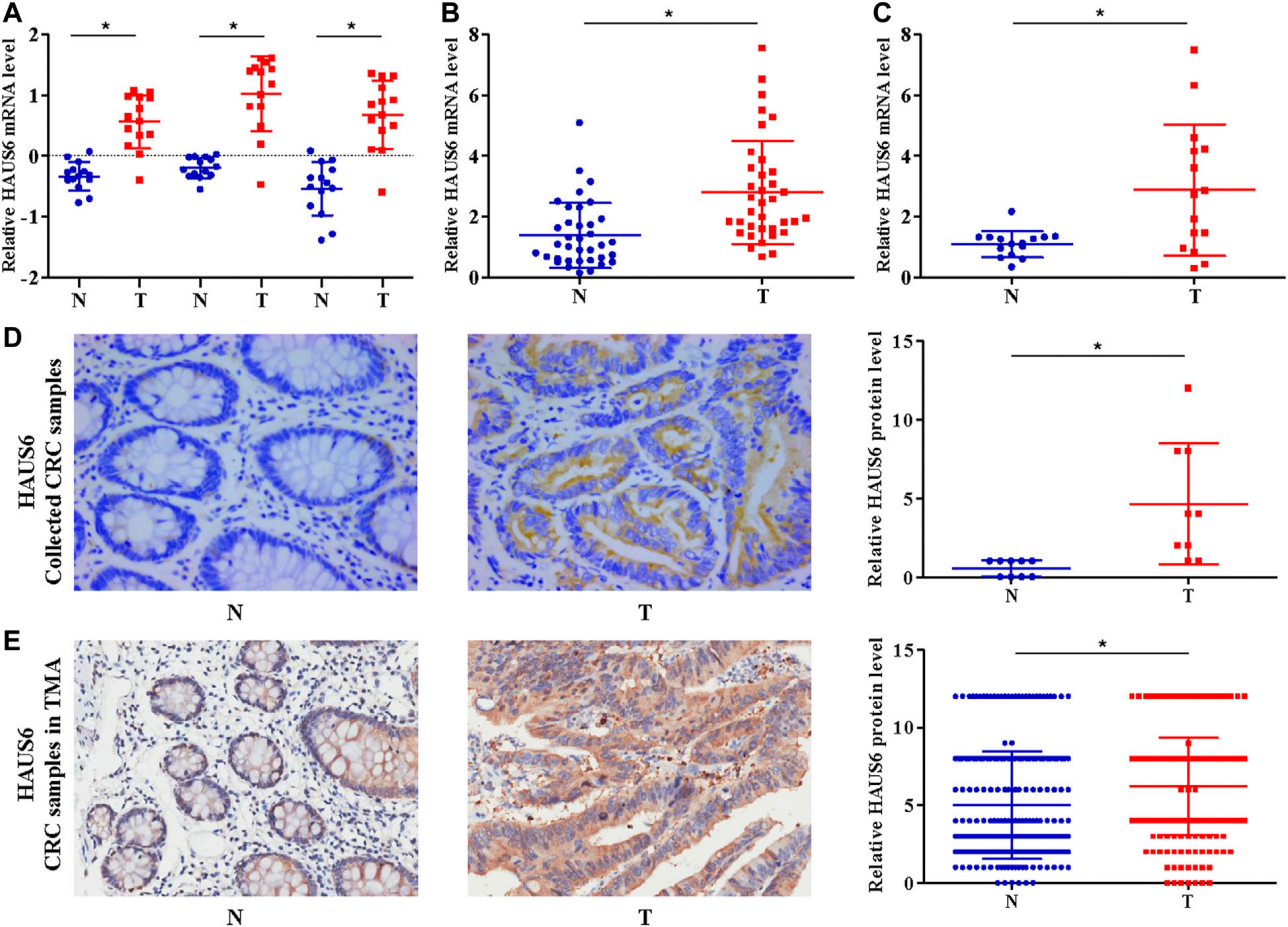
HAUS6 mRNA and protein are up-regulated in CRC tissues. **(A)** HAUS6 expression (3 different probes) in 14 matched CRC and noncancerous colorectal tissues from our previously described gene expression profile microarrays (GEO Submission: GSE113513). Each dot represents 1 tissue; Mean ± SD; *p < 0.05, vs. N, paired Student’s *t* test. **(B,C)** Quantitative RT-PCR determination of HAUS6 mRNA expression in **(B)** 36 matched CRC and noncancerous colorectal tissues collected by our laboratory and **(C)** 15 matched CRC and noncancerous colorectal tissues from commercially available cDNA arrays. GAPDH was used as an internal control. Mean ± SD; *p < 0.05, vs. N, by paired Student’s *t* test. **(D,E)** Immunohistochemistry to determine HAUS6 protein levels in **(D)** 10 matched CRC and noncancerous colorectal tissues collected by our laboratory (Magnification: ×40) and **(E)** 260 matched CRC and noncancerous colorectal tissues from commercially available tissue microarrays (Magnification: ×20). Representative images are shown on the lower panel. Mean ± SD; *p < 0.05, vs. N, by paired Student’s *t* test. T: colorectal cancer tissues; N: noncancerous colorectal tissues.

We first compared HAUS6 expression in cDNA samples from 14 matched pairs of primary CRC tumors and non-cancerous tissues from a cDNA array (GEO Submission: GSE113513). HAUS6 expression was higher in CRC tumors than non-cancerous tissues (Figure 1A). Analysis of publicly available tumor expression data from the Oncomine database (http://www.oncomine.com/) also showed higher HAUS6 mRNA levels in CRC tissues than non-cancerous tissues (Supplementary Table S1). Quantitative RT-PCR analysis of HAUS6 expression in clinical samples of 36 CRC tissues and adjacent non-cancerous colorectal tissues from our laboratory and a cDNA tissue array containing 15 pairs of CRC primary tumors and surrounding non-cancerous tissues also showed higher HAUS6 mRNA expression in CRC tissues (Figures 1B,C). Immunohistochemistry of 10 pairs of CRC samples randomly selected from the above mentioned 36 pairs, as well as analysis of a tissue microarray of 260 pairs of CRC samples (Shanghai Outdo Biotech), confirmed that HAUS6 protein levels were higher in CRC primary tumor tissues (Figures 1D,E).

### HAUS6 Expression is Associated With Poor Patient Prognosis

**FIGURE 2:**
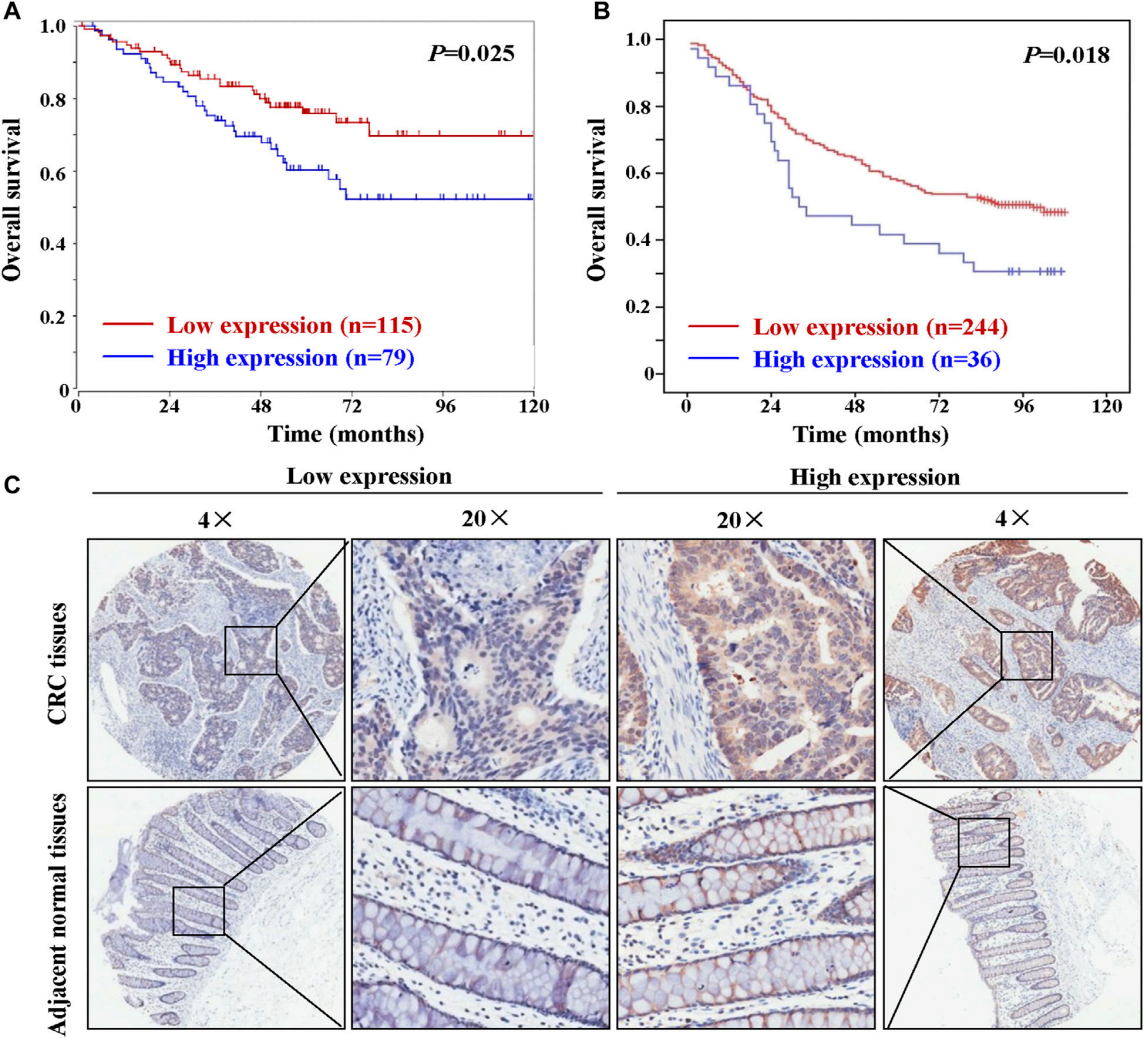
High HAUS6 expression is associated with shorter overall survival in CRC patients. **(A)** Overall survival of 194 CRC patients with low and high HAUS6 mRNA expression (GEO ID: GSE24551), based on data in the public microarray dataset that were analyzed through the R2 bioinformatic platform by Kaplan-Meier using the log-rank test. **(B)** Kaplan-Meier plots showing overall survival of 280 CRC patients with low and high HAUS6 protein expression by Kaplan-Meier using the log-rank test. **(C)** Representative immunohistochemistry images showing high or low HAUS6 expression. Magnification: ×4 and ×20.

Kaplan-Meier analysis of 80 patients whose primary tumor tissues were sampled on the cDNA array mentioned above did not reveal any difference in patient survival between tumors with low and high HAUS6 mRNA expression (data not shown). In contrast, analysis of data from 194 CRC patients (high expression: n = 79; low expression: n = 115) from the public microarray dataset using the R2 bioinformatic platform showed that high HAUS6 mRNA expression was associated with shorter overall survival (Figure 2A). Similarly, tumors with high HAUS6 protein levels were associated with shorter overall survival in 280 CRC patients (Figure 2B; high expression: n = 36; low expression: n = 244). Representative images of high or low expression of HAUS6 in CRC tissues are shown in Figure 2C.

### HAUS6 Knockdown inhibits CRC Cell Growth and Proliferation *in Vitro*

**FIGURE 3:**
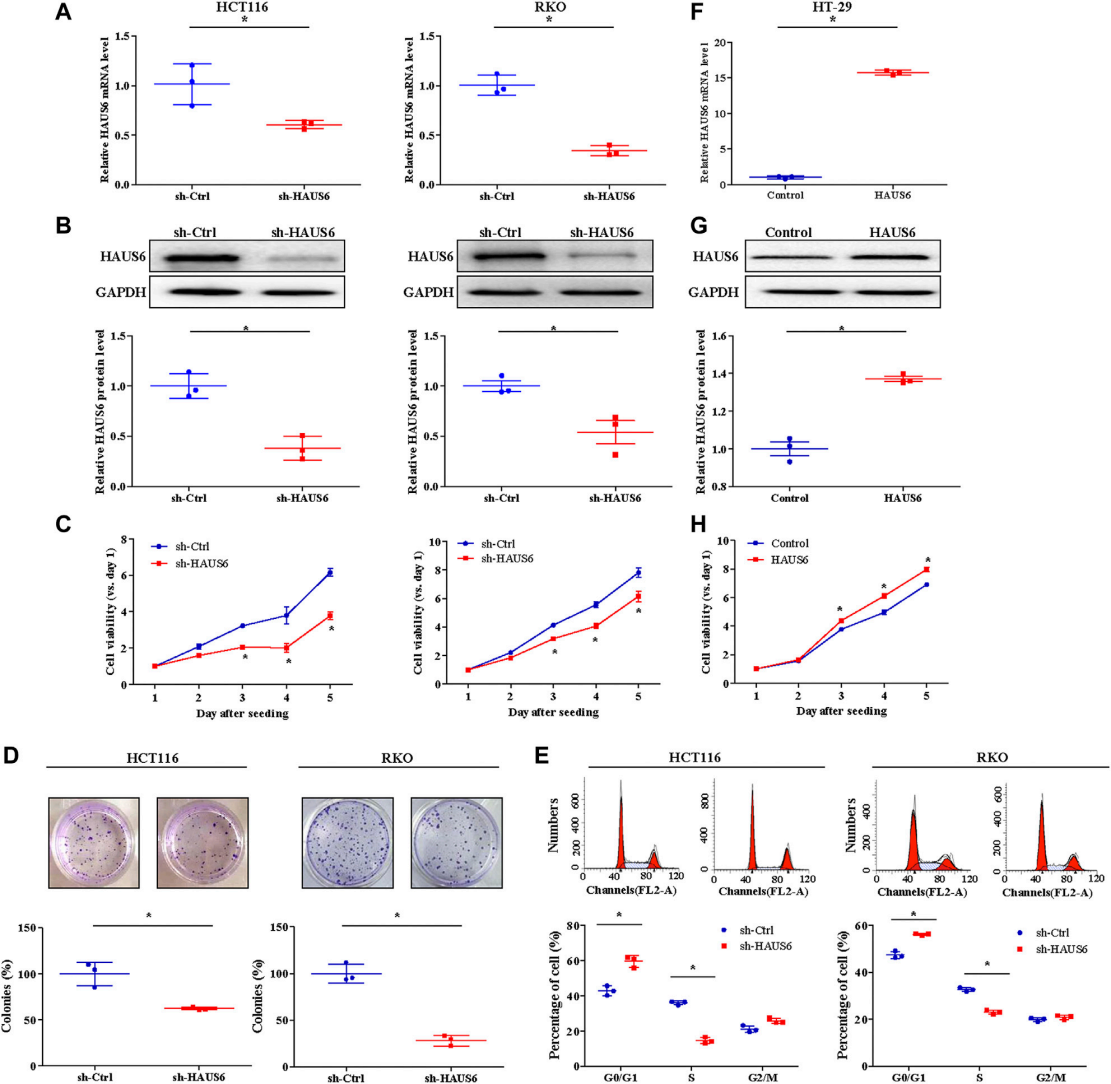
HAUS6 knockdown reduces CRC cell growth and proliferation *in vitro.* **(A)** The mRNA and **(B)** protein levels of HAUS6 in HCT116 (left panel) and RKO (right panel) cells after transduction of sh-HAUS6 or sh-Ctrl were assessed by quantitative RT-PCR and western blotting (n = 3). GAPDH was used as a loading control. Band intensities were quantified using ImageLab software. Mean ± SD; *p < 0.05, vs. sh-Ctrl, by independent Student’s *t* test. **(C)** Viability of HCT116 (left panel) and RKO (right panel) cells after knockdown of HAUS6 was determined by CCK-8 assay. Data are normalized to viability on day 1 and are represented as fold changes. Mean ± SD; n = 6; *p < 0.05, vs. sh-Ctrl, by independent Student’s *t* test. **(D)** Survival of HCT116 (left panel) and RKO (right panel) cells transduced with shRNA against HAUS6 or a control shRNA was assessed in the colony formation assay. Representative images of colonies are shown above the graphs. Cell survival was normalized to the sh-Ctrl group. Mean ± SD; n = 3; *p < 0.05, vs. sh-Ctrl, by independent Student’s *t* test. **(E)** Percentage of HCT116 (left panel) and RKO (right panel) cells transduced with shRNA against HAUS6 or a control shRNA in G0/G1, S, and G2/M phases was assessed by flow cytometry. Representative flow cytometry plots are shown above, and the percentages of cells in each phase are shown below. Mean ± SD; n = 3; *p < 0.05, vs. sh-Ctrl, by independent Student’s *t* test. **(F)** The mRNA and **(G)** protein levels of HAUS6 in HT-29 cells after transduction of lentivirus encoding HAUS6 or Control plasmid were assessed by quantitative RT-PCR and western blotting (n = 3). GAPDH was used as a loading control. Band intensities were quantified using ImageLab software. Mean ± SD; *p < 0.05, vs. sh-Ctrl, by independent Student’s *t* test. **(H)** Viability of HT-29 cells after HAUS6 over-expression was determined by CCK-8 assay. Data are normalized to viability on day 1 and are represented as fold changes. Mean ± SD; n = 6; *p < 0.05, vs. Control, by independent Student’s *t* test.

In cultured RKO and HCT116 cells, which constitutively express relatively high levels of endogenous HAUS6 (Supplementary Figure S1A), we assessed the effects of HAUS6 knockdown on growth and proliferation of CRC cells. Lentiviral transduction with vectors expressing short hairpin (sh)RNAs against HAUS6 down-regulated HAUS6, as expected (Figures 3A,B; Supplementary Figures S1B–C). CRC cells transduced with HAUS6 shRNAs had lower cell viability than those transduced with control RNAs (Figure 3C; Supplementary Figure S1D). Moreover, HAUS6 knockdown also decreased the number of colonies formed by CRC cells (Figure 3D). Cell cycle analysis revealed that HAUS6 knockdown increased the percentage of cells in G0/G1 and decreased the percentage of cells in S phase (Figure 3E). However, HAUS6 knockdown did not affect cell apoptosis in HCT116 cells (Supplementary Figure S2). In contrast, in HT-29 cells which exhibits relative lower expression of HAUS6 (Supplementary Figure S1A), lentiviral transduction with vectors over-expressing HAUS6 up-regulated HAUS6 protein expression, and increased the cell viability of HT-29 cells based on the CCK8 assay (Figures 3F–H).

### HAUS6 Knockdown Suppresses CRC Cell Growth and Proliferation *in Vivo*

**FIGURE 4:**
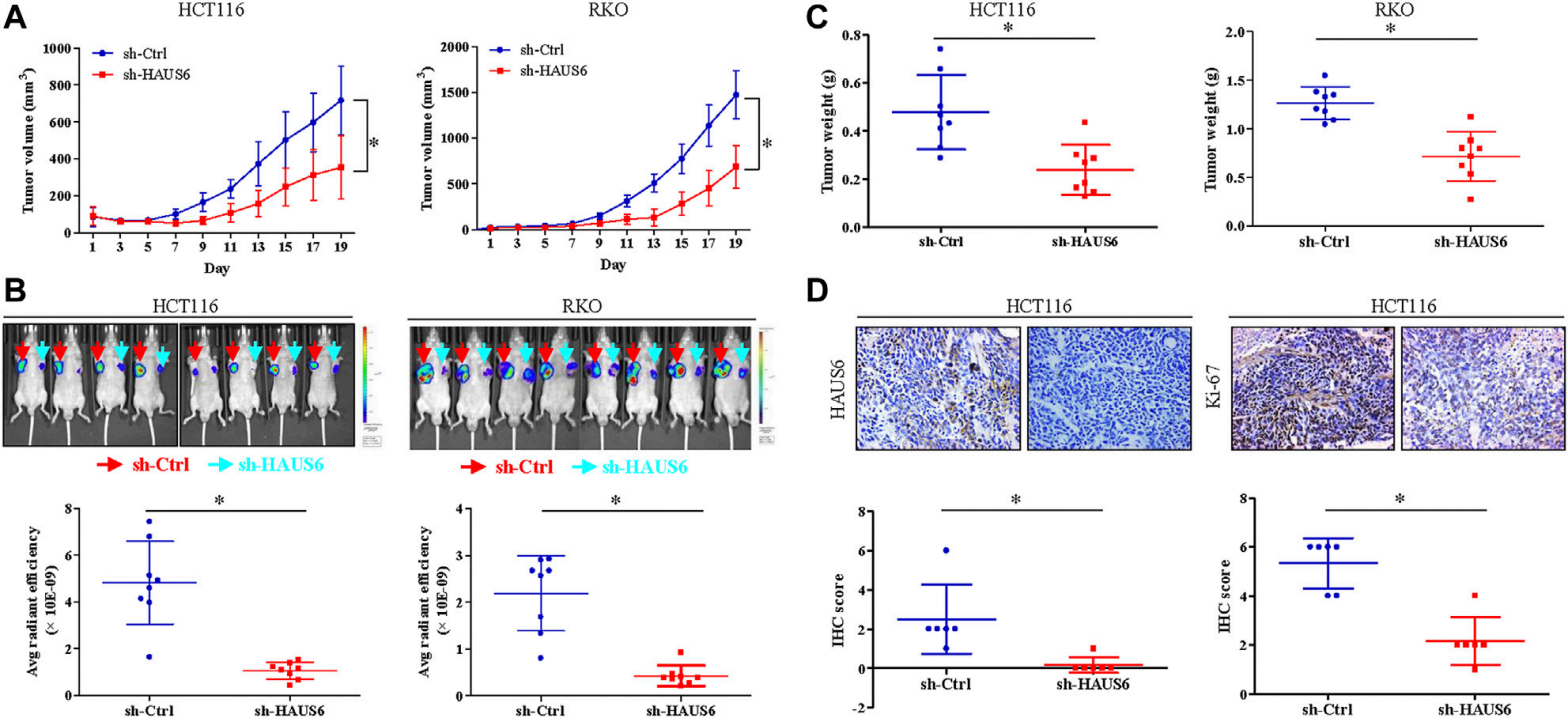
HAUS6 knockdown reduces tumor growth and cell proliferation *in vivo.* **(A)** Tumor volume in BALB/c nude mice injected with HCT116 (left panel) or RKO (right panel) cells transduced with shRNA against HAUS6 or control shRNA. Mean ± SD; n = 8; *p < 0.05, vs. sh-Ctrl, by independent Student’s *t* test. **(B)** GFP fluorescence in BALB/c nude mice injected with HCT116 (left panel) or RKO (right panel) cells transduced with a GFP reporter and shRNA against HAUS6 or control shRNA. Representative fluorescence images are shown above, and quantitation of GFP intensity is shown below. Mean ± SD; n = 8; *p < 0.05, vs. sh-Ctrl, by independent Student’s *t* test. **(C)** Tumor weight in BALB/c nude mice injected with HCT116 (left panel) or RKO (right panel) cells transduced with shRNA against HAUS6 or control shRNA on day 26 after injection. Mean ± SD; n = 8; *p < 0.05, vs. sh-Ctrl, by independent Student’s *t* test. **(D)** HAUS6 and Ki-67 protein levels in excised tumors from BALB/c nude mice injected with HCT116 cells transduced with shRNA against HAUS6 or control shRNA. Representative immunohistochemistry images are shown above. Magnification: ×40; Mean ± SD; n = 6; *p < 0.05, vs. sh-Ctrl, by independent Student’s *t* test.

We next used a xenograft mouse model to assess the effects of HAUS6 knockdown on CRC cell growth and proliferation *in vivo*. HAUS6 knockdown strongly reduced tumor volume (Figure 4A) and decreased fluorescence of tumor cells (Figure 4B) in nude mice injected with GFP-expressing CRC cells. Final tumor weights were also lower in mice injected with HAUS6 knockdown cells than in those injected with control cells (Figure 4C). Immunohistochemistry of the excised tumors showed that Ki-67 protein expression was decreased in HAUS6 knockdown cells (Figure 4D), suggesting that HAUS6 knockdown suppresses CRC tumor growth by inhibiting cell proliferation.

### HAUS6 Knockdown Suppresses Cell Proliferation by Down-Regulating p21

**FIGURE 5:**
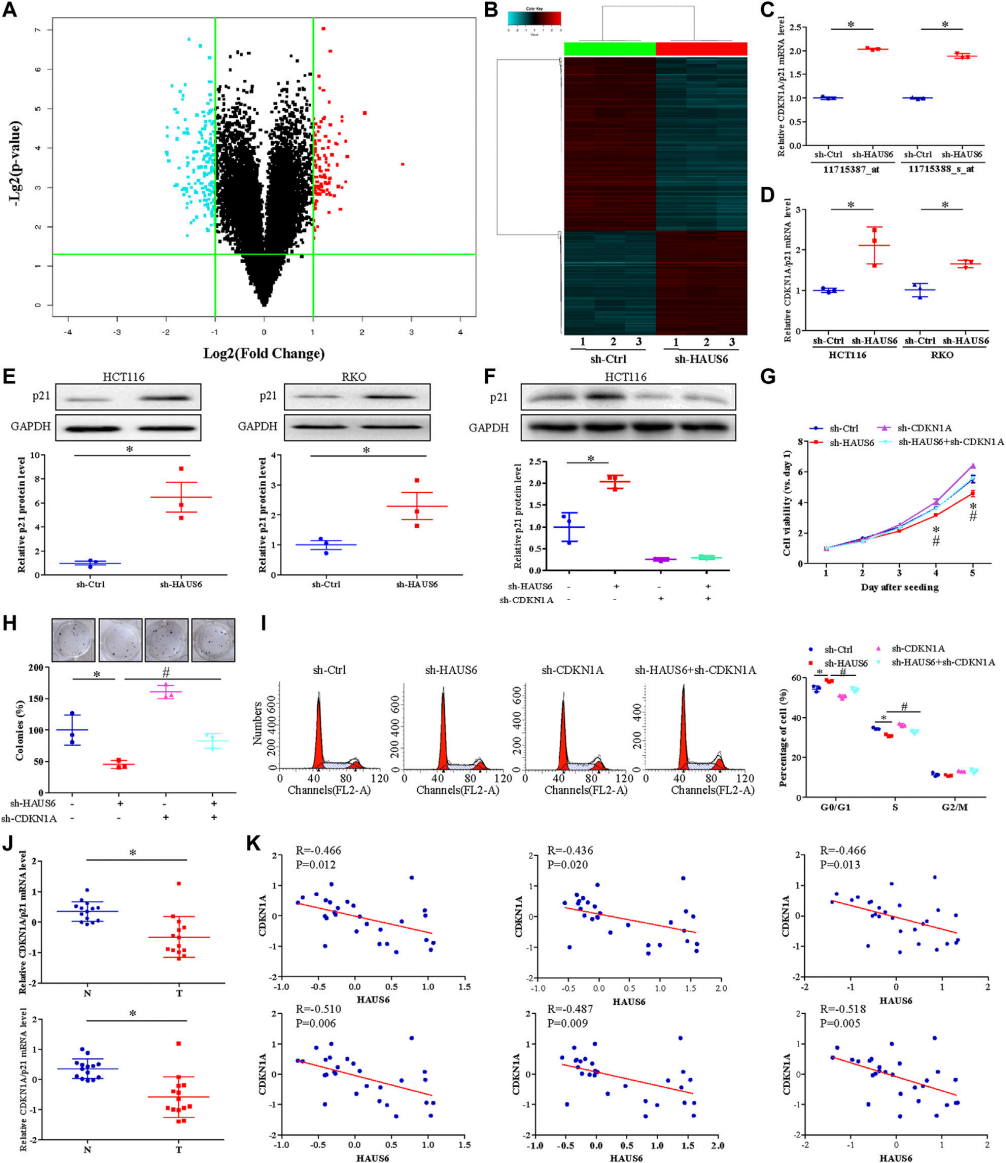
HAUS6 knockdown suppresses cell proliferation by increasing p21 expression. **(A)** A volcano plot and **(B)** hierarchical clustering plot showing DEGs (fold change >2, *p* < 0.05) between HCT116 cells transduced with shRNA against HAUS6 or control shRNA (n = 3). **(C,D)** CDKN1A/p21 mRNA levels was assessed by **(C)** cDNA microarray in HCT116 cells transduced with shRNA against HAUS6 or control shRNA and **(D)** quantitative RT-PCR in HCT116 and RKO cells transduced with shRNA against HAUS6 or control shRNA. GAPDH was used as an internal control. Mean ± SD; n = 3; *p < 0.05, vs. sh-Ctrl by independent Student’s *t* test. **(E)** Protein levels of p21 in HCT116 and RKO cells after HAUS6 knockdown was assessed by western blot (n = 3). GAPDH was used as a loading control. Band intensities were quantified using ImageLab software. Mean ± SD; n = 3; *p < 0.05, vs. sh-Ctrl by independent Student’s *t* test. **(F)** Expression of p21 protein in HCT116 cells transduced with shRNAs against HAUS6, CDKN1A or both, or control shRNA was assessed by western blot (n = 3). GAPDH was used as a loading control. Band intensities were quantified using ImageLab software. Mean ± SD; n = 3; *p < 0.05, vs. sh-Ctrl and #*p* < 0.05, vs. sh-HAUS6, by one-way ANOVA with LSD post hoc test. **(G)** Viability of HCT116 cells transduced with shRNA against HAUS6, CDKN1A, or both, or control shRNA. Data were normalized to day 1 and presented as fold changes. Mean ± SD; n = 6; *p < 0.05, vs. sh-Ctrl and #*p* < 0.05, vs. sh-HAUS6, by one-way ANOVA with LSD post hoc test. **(H)** Cell survival was assessed by colony formation assay. Representative images of colonies (upper panel) and numbers of colonies (lower panel) are shown. The number of colonies was normalized to the sh-Ctrl group. Mean ± SD; n = 3; *p < 0.05, vs. sh-Ctrl and #*p* < 0.05, vs. sh-HAUS6, by one-way ANOVA with LSD post hoc test. **(I)** Percentages of HCT116 cells transduced with shRNA against HAUS6, CDKN1A, or both, or control shRNA that were in G0/G1, S, or G2/M phase was determined by flow cytometry. Representative flow cytometry plots are shown on the left. Mean ± SD; n = 3; *p < 0.05, vs. sh-Ctrl and #*p* < 0.05, vs. sh-HAUS6, by one-way ANOVA with LSD post hoc test. **(J)** CDKN1A mRNA levels in 14 matched pairs of CRC and non-cancerous tissues was assessed by two microarray probes (n = 14). **(K)** Pearson’s rank correlation analysis between HAUS6 expression (three microarray probes) and CDKN1A expression (two microarray probes) in CRC tissues.

Microarray analysis of HAUS6 knockdown HCT116 cells revealed a total of 274 differentially expressed genes (DEGs) in CRC (Figures 5A,B; GEO Submission: GSE140326). CDKN1A (encoding p21 protein) was upregulated in HAUS6 knockdown cells (Figure 5C), and this was confirmed in both HCT116 and RKO cells by quantitative RT-PCR (Figure 5D) and western blotting (Figure 5E). Transduction with shRNAs specific for HAUS6 significantly up-regulated p21 protein expression, which were reversed after CDKN1A knockdown in HCT116 cells (Figure 5F). Functionally, HAUS6 knockdown decreased cell viability and colony formation, whereas CDKN1A knockdown had the opposite effect and attenuated the effects of HAUS6 knockdown (Figures 5G,H). HAUS6 knockdown increased the percentage of cells in G0/G1 and reduced the percentage in S phase, which were reversed by CDKN1A knockdown (Figure 5I). Analysis of microarray expression data (GEO Submission: GSE113513) showed that CDKN1A was down-regulated in CRC tissues (Figure 5J) and its expression correlated negatively with HAUS6 expression (Figure 5K).

### HAUS6 Knockdown Suppresses Cell Proliferation by Activating the p53 Pathway

**FIGURE 6:**
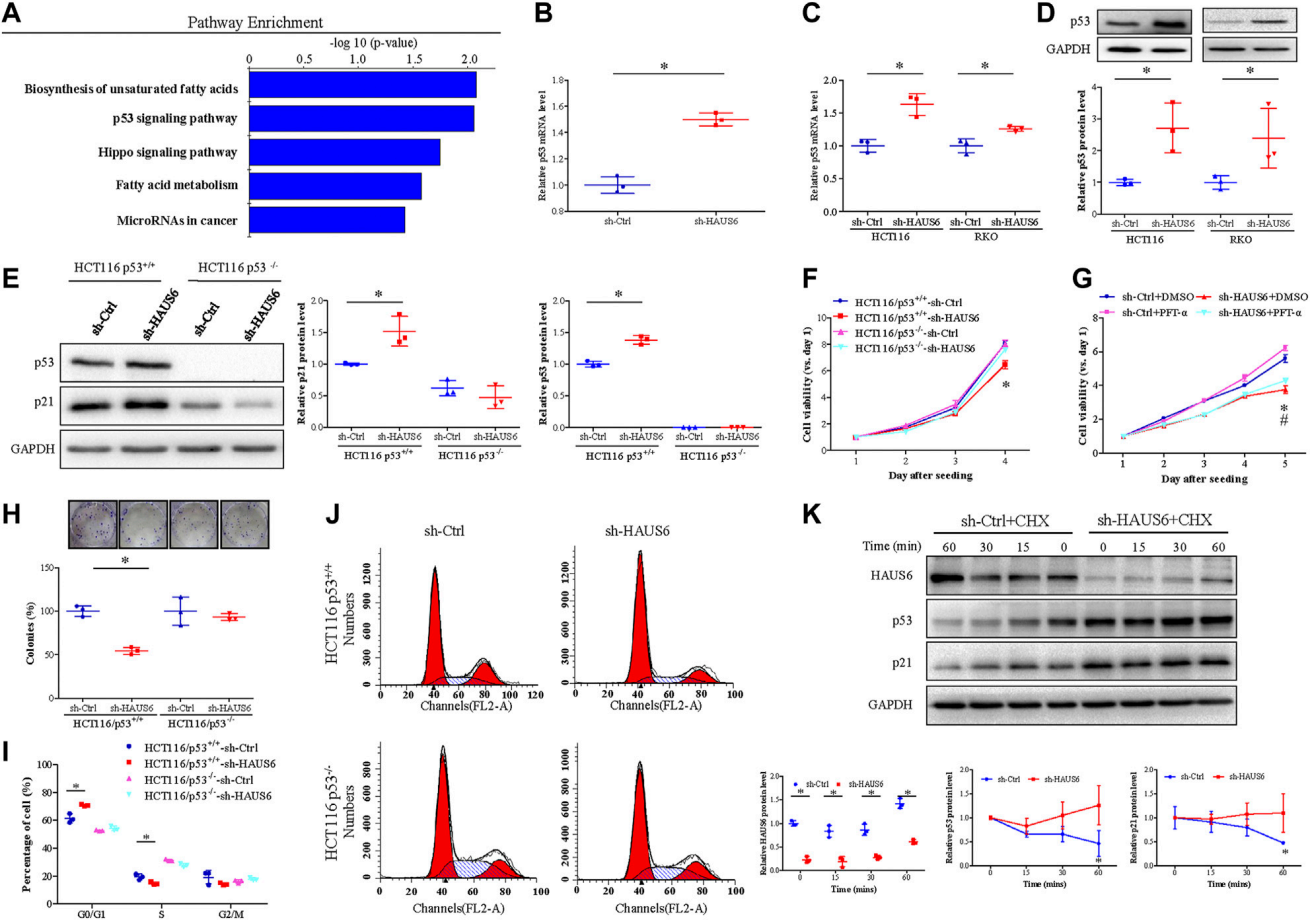
HAUS6 knockdown inhibits cell proliferation by activating the p53/p21 pathway. **(A)** The top five pathways enriched in DEGs was identified by KEGG pathway enrichment analysis (all *p* < 0.05). **(B-C)** TP53 mRNA levels in cells transduced with shRNA against HAUS6 or control shRNA was assessed by **(B)** microarray (HCT116 cells) and **(C)** quantitative RT-PCR (HCT116 and RKO cells). Mean ± SD; n = 3; *p < 0.05, vs. sh-Ctrl, by independent Student’s *t* test. GAPDH was used as an internal control. **(D)** Protein levels of p53 in HCT116 and RKO cells after HAUS6 knockdown was detected by western blot. GAPDH was used as a loading control. Band intensities were quantified using ImageLab software. Mean ± SD; n = 3; *p < 0.05, vs. sh-Ctrl by independent Student’s *t* test. **(E)** Protein levels of p53 in HCT116/p53^+/+^ and HCT116/p53^−/−^ cells after HAUS6 knockdown was assessed by western blot. GAPDH was used as a loading control. Band intensities were quantified using ImageLab software. Mean ± SD; n = 3; *p < 0.05, vs. sh-Ctrl of HCT116/p53^+/+^ cells, by independent Student’s *t* test. **(F)** Viability of HCT116/p53^+/+^ cells and HCT116/p53^−/−^ cells transduced with shRNA against HAUS6 or control shRNA. Results were normalized to viability on day 1. Mean ± SD; n = 6; *p < 0.05, vs. sh-Ctrl of HCT116/p53^+/+^ cells, by independent Student’s *t* test. **(G)** Cell viability in HCT116 cells treated with p53 inhibitor. Results were normalized to viability on day 1. Mean ± SD; n = 6; *p < 0.05, vs. sh-Ctrl and #*p* < 0.05, vs. sh-HAUS6+DMSO by one-way ANOVA with LSD post hoc test. **(H)** Survival of HCT116/p53^+/+^ cells and HCT116/p53^−/−^ cells transduced with shRNA against HAUS6 or control shRNA. Representative images of colonies are shown in the upper panel. Cell survival was normalized relative to the sh-Ctrl group. Mean ± SD; n = 3; *p < 0.05, vs. sh-Ctrl of HCT116/p53^+/+^ cells, by independent Student’s *t* test. **(I–J)** Cell cycle distribution of HCT116/p53^+/+^ and HCT116/p53^−/−^ cells transduced with shRNA against HAUS6 or control shRNA were determined by FACS analysis, **(I)** the representative flow cytometry plots were shown. **(J)** The percentages of cells in G0/G1, S, and G2/M phases were analyzed. Mean ± SD; n = 3; *p < 0.05, vs. sh-Ctrl of HCT116/p53^+/+^ cells, by independent Student’s *t* test. **(K)** HAUS6, p53 and p21 protein levels in RKO cells transduced with shRNA against HAUS6 or control shRNA after cycloheximide (CHX) treatment was determined by western blot (n = 3). GAPDH was used as a loading control. Band intensities were quantified using ImageLab software. Mean ± SD; n = 3; *p < 0.05, vs. sh-Ctrl; by independent Student’s *t* test.

KEGG pathway enrichment analysis of DEGs in HAUS6 knockdown cells identified the p53 pathway as the one of the most enriched signalling pathways (Figure 6A). Given the key regulatory effects of p53 on cell proliferation and CDKN1A transcription, we next explored the effect of HAUS6 on the p53 pathway. HAUS6 knockdown increased TP53 expression in HCT116 and RKO cells at the levels of mRNA (Figures 6B,C) and protein (Figure 6D). The inhibitory effects of HAUS6 knockdown on cell viability were attenuated by knockout of p53 (Figures 6E,F) or treatment with the p53 inhibitor PFT-α (Figure 6G). Similarly, p53 knockout blocked the inhibitory effects of HAUS6 knockdown on cell survival (Figure 6H) and cell cycle progression (Figures 6I,J). To determine the molecular mechanism by which HAUS6 knockdown regulates p53, we evaluated p53 protein stability in RKO cells expressing wild-type p53. Protein levels of p53 and its downstream p21 decreased over time in sh-Ctrl cells, but remained stable after HAUS6 knockdown (Figure 6K).

### HAUS6 Knockdown Enhances 5- Fluorouracil Treatment on Suppressing Cell Proliferation by Activating the p53/p21 Pathway

**FIGURE 7:**
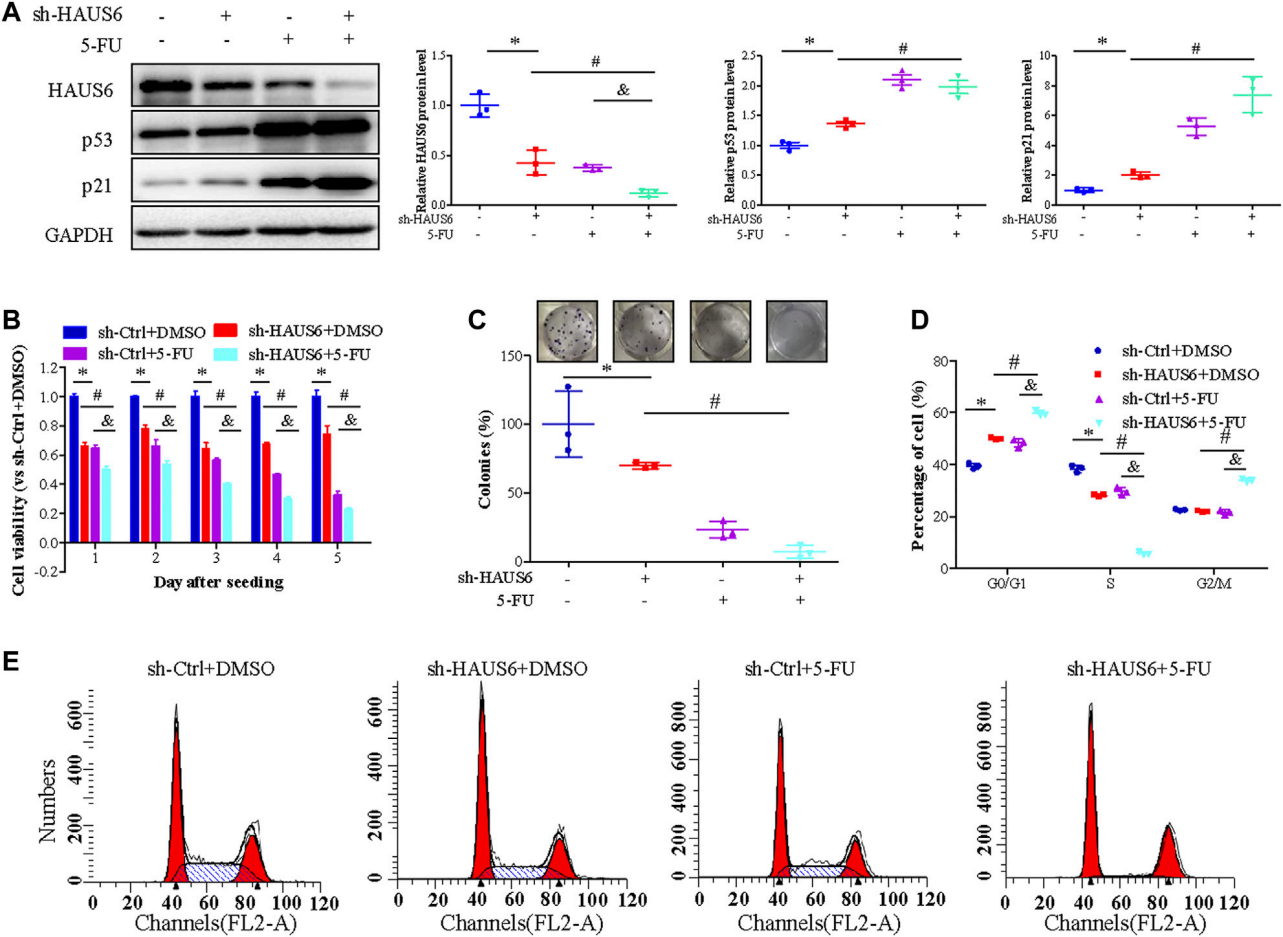
5-FU treatment enhances HAUS6 knockdown on suppressing CRC cell growth by activating the p53/p21 pathway. **(A)** HAUS6, p53 and p21 protein levels in HCT116 cells transduced with shRNA against HAUS6 or control shRNA and treated with 5-FU, was determined by western blot. GAPDH was used as a loading control. Band intensities were quantified using ImageLab software. Mean ± SD; n = 3; *p < 0.05, vs. sh-Ctrl, #*p* < 0.05 vs. sh-HAUS6+DMSO, &*p* < 0.05, vs. sh-Ctrl+5-FU, by one-way ANOVA with LSD post hoc test. **(B)** Viability of HCT116 cells transduced with shRNA against HAUS6 or control shRNA and treated with 2.5 μM of 5-FU for 24h, was determined by CCK-8 assay. Results were normalized to the sh-Ctrl group. Mean ± SD; n = 6; *p < 0.05, vs. sh-Ctrl, #*p* < 0.05 vs. sh-HAUS6+DMSO, &*p* < 0.05, vs. sh-Ctrl+5-FU, by one-way ANOVA with LSD post hoc test. **(C)** Survival of HCT116 cells transduced with shRNA against HAUS6 or control shRNA and treated with 5-FU, was assessed by colony formation assay. Representative images of colonies are shown on the upper panel, and cell survival normalized to the sh-Ctrl group is shown on the lower panel. Mean ± SD; n = 3; *p < 0.05, vs. sh-Ctrl, #*p* < 0.05 vs. sh-HAUS6+DMSO, &*p* < 0.05, vs. sh-Ctrl+5-FU, by one-way ANOVA with LSD post hoc test. **(D,E)** Cell cycle distribution of HCT116 cells transduced with shRNA against HAUS6 or control shRNA and treated with 5-FU, was assessed by flow cytometry. **(D)** The representative flow cytometry plots are shown. **(E)** The percentages of cells in G0/G1, S, and G2/M phase are shown. Mean ± SD; n = 3; *p < 0.05, vs. sh-Ctrl, #*p* < 0.05 vs. sh-HAUS6+DMSO, &*p* < 0.05, vs. sh-Ctrl+5-FU, by one-way ANOVA with LSD post hoc test.

We further assessed the effect of HAUS6 on the response of CRC cells to 2.5 μM 5-fluorouracil (5-FU) treatment for 24 h. Transduction of sh-HAUS6 or 5-FU treatment down-regulated HAUS6 and up-regulated p53 and p21 protein expression. These effects of HAUS6 knockdown were further enhanced after combined with 5-FU treatment (Figure 7A). Moreover, HAUS6 knockdown or 5-FU treatment reduced viability of HCT116 cells, and combination of them exhibited greater suppression on cell viability of HCT116 cells at different time points (Figure 7B). Moreover, combining HAUS6 knockdown with 5-FU treatment led to greater suppression of HCT116 colony formation than HAUS6 knockdown or 5-FU treatment alone (Figure 7C), as well as higher percentages of cells in G0/G1 and G2/M and lower percentage in S phase (Figures 7D,E).

## Discussion

In this study, we show that the spindle-associated protein HAUS6 has an oncogenic role in CRC. HAUS6 mRNA and protein expression were both increased in CRC tissues compared to adjacent noncancerous colorectal tissues, and higher HAUS6 expression was correlated with shorter overall survival in CRC patients. HAUS6 knockdown suppressed tumor growth by inhibiting cell viability and survival as well as by arresting cells in G0/G1, which were enhanced after combination of 5-FU treatment. HAUS6 knockdown exerted these effects by reducing p53 degradation and activating the p53/p21 signaling pathway.

For the first time, we evaluated the clinic significance of HAUS6 in CRC. Analysis from our own cDNA array (Shen et al., 2019) found that HAUS6 mRNA expression was increased in CRC tissues compared to adjacent noncancerous colorectal tissues. This was consistent with our analysis of multiple datasets from the Oncomine database, quantitative RT-PCR analysis of a cDNA tissue array, and immunohistochemical analysis of a tissue microarray. These results suggest that increased HAUS6 expression may be common in CRC and may play an essential role in CRC development. Moreover, survival analysis revealed a correlation between high HAUS6 mRNA and protein expression and shorter overall survival in CRC patients. This demonstrates that HAUS6 may be useful as a biomarker for CRC prognosis. However, HAUS6 expression should first be further investigated in other types of malignancies.

Due to the potential significantly value of HAUS6 in development and therapy of CRC, it’s urgent need to investigate the biological function of HAUS6 in CRC. However, the biological function of HAUS6 in most malignant neoplasms (including CRC) remain largely unknown. Cancer cells are characterized by an uncontrolled increase in cell proliferation (Sherr, 1996; Yuba-Kubo et al., 2005), which requires mitotic spindle formation. Nucleation of MTs is the initial step of mitotic spindle formation (Holland and Cleveland, 2012; Nakayama et al., 2013), and loss-of-function in MT-dependent MT amplification results in mitotic arrest (Jang et al., 2016). Therefore, inhibition of mitotic spindle formation by targeting microtubule nucleation factors may be a promising anti-cancer strategy (Karna et al., 2011; Bates and Eastman, 2017; Kollareddy et al., 2017; Paier et al., 2018). Many microtubule inhibitors, e.g. taxans, vinca alkaloids and paclitaxel, induce mitotic arrest by interfering with microtubule dynamics and have been used to treat cancer (Taylor et al., 2008; Ehrhardt et al., 2013; Po’uha and Kavallaris, 2015). However, due to side effects in normal cells and acquired resistance in cancer cells, it is important to further explore the underlying mechanism. Series of functional study show that knockdown of the spindle assembly factor HAUS6 suppressed tumor growth *in vivo* and *in vitro* by inhibiting cell viability, survival and cell cycle progression. In contrast, HAUS6 over-expression obviously increased cell viability of HCT116 cells *in vitro.* We further show that HAUS6 knockdown enhanced the ability of 5-FU to decrease cell viability and survival and to arrest cells in G0/G1 and G2/M phases. These results suggest that HAUS6 may be a promising new target for anticancer treatments.

The potential of HAUS6 serving as a therapy target encouraged us to further explore its underlying mechanism on suppression cell growth in CRC. Microarray analysis of HAUS6 knockdown cells revealed 103 up-regulated genes and 171 down-regulated genes. Many of them, including CDKN1A (Lodygin et al., 2002), CyclinD1 (Wang et al., 2018), ROCK2 (Li et al., 2017) and HMGA2 (Gao et al., 2017), have already been implicated in proliferation and cell cycle progression. One notable DEG was CDKN1A (p21), which arrests cell cycle progression at G1/S phase by binding to and inhibiting cyclin-dependent kinases (Harper et al., 1993). We found that p21 protein levels were increased in HAUS6 knockdown cells, and that CDKN1A knockdown reversed the effects of HAUS6 knockdown on cell viability, cell survival and cell cycle arrest. Moreover, HAUS6 expression was inversely correlated with CDKN1A expression. These results indicate that CDKN1A may mediate the oncogenic effects of HAUS6. However, the regulatory effects of HAUS6 on p21 need to be further explored.

To further explore its regulatory effects on related signalling pathway, KEGG pathway enrichment analysis of DEGs in HAUS6 knockdown cells showed that the p53 pathway was one of the most enriched. The p53 protein upregulates p21 in response to stress stimuli by binding to two highly conserved p53 response elements in the p21 promoter (el-Deiry et al., 1993; Jung et al., 2010). As a tumor suppressor, p53 is expressed at low levels under normal conditions due to MDM2, which mediates nuclear export of p53 and targets p53 for ubiquitination and degradation. Under stress conditions, p53 rapidly accumulates and activates p21 (Wade Harper et al., 1993; el-Deiry et al., 1993; Vogelstein et al., 2000). In our experiments, HAUS6 knockdown increased TP53 mRNA and protein expression. Knockout of p53 also abrogated the effects of HAUS6 knockdown on cell viability, survival and cell cycle arrest in HCT116 cells. Therefore, the suppression of tumor growth caused by HAUS6 knockdown depends at least partly on activation of p53. We further found that HAUS6 knockdown reduced the degradation of p53 and p21, suggesting a mechanism by which HAUS6 regulates p53.

Taken together, these results show that HAUS6 knockdown suppresses CRC tumor growth by increasing the stability of p53. Moreover, combining HAUS6 knockdown with 5-FU treatment led to even greater down-regulation of HAUS6 and up-regulation of p21 and p53 than HAUS6 knockdown alone, suggesting a common therapeutic mechanism. The effect of HAUS6 on tumorigenesis and p53 expression is similar to that of Plk1, which recruits HAUS6 to spindle MTs and NEDD1 to the spindle and centrosomes during mitosis (Zhu et al., 2008). HAUS6 may therefore be a promising novel target for anticancer treatments. Further study should be done to investigate the mechanism by which HAUS6 knockdown activates the p53 pathway.

In summary, we show that HAUS6 upregulation is associated with shorter survival of CRC patients and increased cell viability in cultured CRC cells, while HAUS6 knockdown inhibits tumor growth and enhances 5-FU treatment by activating the p53/p21 pathway. HAUS6 may be a useful prognostic marker and chemotherapeutic target in CRC.

## Data Availability

The datasets presented in this study can be found in online repositories. The names of the repository/repositories and accession number(s) can be found below: NCBI [accession: GSE140326].

## Abbreviations

CRC, colorectal cancer; EGFP, enhanced green fluorescent protein; FJTCM, Fujian University of Traditional Chinese Medicine; FACS, fluorescence activated cell sorting; HAUS6, HAUS augmin like complex subunit 6; MT, microtubule; NEDD1, neural precursor cell-expressed developmentally down-regulated protein 1; RT-PCR, quantitative reverse transcription - polymerase chain reaction; 5-FU, 5-fluorouracil; p-TNM, pathologic tumor-node-metastasis; TMA, tissue microarray; SPF, specific pathogen-free.

## References

[B1] Bates D. Eastman A. (2017). Microtubule Destabilising Agents: Far More Than Just Antimitotic Anticancer Drugs. Br. J Clin Pharmacol 83 (2), 255–268. 10.1111/bcp.13126 27620987 PMC5237681

[B2] Chouinard G. Clément I. Lafontaine J. Rodier F. Schmitt E. (2013). Cell Cycle-dependent Localization of CHK2 at Centrosomes during Mitosis. Cell Div 8 (1), 7. 10.1186/1747-1028-8-7 23680298 PMC3668180

[B3] Du L. Xu J. Li X. Ma N. Liu Y. Peng J. (2011). Rumba and Haus3 Are Essential Factors for the Maintenance of Hematopoietic Stem/progenitor Cells during Zebrafish Hematopoiesis. Development (Cambridge, England) 138 (4), 619–629. 10.1242/dev.054536 21228005

[B4] Ehrhardt H. Pannert L. Pfeiffer S. Wachter F. Amtmann E. Jeremias I. (2013). Enhanced Anti-tumour Effects of Vinca Alkaloids Given Separately from Cytostatic Therapies. Br. J. Pharmacol. 168 (7), 1558–1569. 10.1111/bph.12068 23186127 PMC3605866

[B5] el-Deiry W. Tokino T. Velculescu V. E. Levy D. B. Parsons R. Trent J. M. (1993). WAF1, a Potential Mediator of P53 Tumor Suppression. Cell 75 (4), 817–825. 10.1016/0092-8674(93)90500-p 8242752

[B6] Fakih M. G. (2015). Metastatic Colorectal Cancer: Current State and Future Directions. J. Clin. Oncol. 33 (16), 1809–1824. 10.1200/jco.2014.59.7633 25918280

[B7] Gao X. Dai M. Li Q. Wang Z. Lu Y. Song Z. (2017). HMGA2 Regulates Lung Cancer Proliferation and Metastasis. Thorac. Cancer 8 (5), 501–510. 10.1111/1759-7714.12476 28752530 PMC5582513

[B8] Goshima G. Mayer M. Zhang N. Stuurman N. Vale R. D. (2008). Augmin: a Protein Complex Required for Centrosome-independent Microtubule Generation within the Spindle. J. Cel. Biol. 181 (3), 421–429. 10.1083/jcb.200711053 PMC2364697 18443220

[B9] Goshima G. Scholey J. M. (2010). Control of Mitotic Spindle Length. Annu. Rev. Cel Dev. Biol. 26, 21–57. 10.1146/annurev-cellbio-100109-104006 20604709

[B10] Hayward D. Metz J. Pellacani C. Wakefield J. G. (2014). Synergy between Multiple Microtubule-Generating Pathways Confers Robustness to Centrosome-Driven Mitotic Spindle Formation. Dev. Cel. 28 (1), 81–93. 10.1016/j.devcel.2013.12.001 PMC3898610 24389063

[B11] Holland A. J. Cleveland D. W. (2012). Losing Balance: the Origin and Impact of Aneuploidy in Cancer. EMBO Rep. 13 (6), 501–514. 10.1038/embor.2012.55 22565320 PMC3367240

[B12] Huang S. Tang R. Poon R. Y. C. (2016). BCL-W Is a Regulator of Microtubule Inhibitor-Induced Mitotic Cell Death. Oncotarget 7 (25), 38718–38730. 10.18632/oncotarget.9586 27231850 PMC5122423

[B13] Jang S. H. Kim A.-R. Park N.-H. Park J. W. Han I.-S. (2016). DRG2 Regulates G2/M Progression via the Cyclin B1-Cdk1 Complex. Mol. Cell 39 (9), 699–704. 10.14348/molcells.2016.0149 PMC5050535 27669826

[B14] Jung Y.-S. Qian Y. Chen X. (2010). Examination of the Expanding Pathways for the Regulation of P21 Expression and Activity. Cell Signal. 22 (7), 1003–1012. 10.1016/j.cellsig.2010.01.013 20100570 PMC2860671

[B15] Karna P. Rida P. C. G. Pannu V. Gupta K. K. Dalton W. B. Joshi H. (2011). A Novel Microtubule-Modulating Noscapinoid Triggers Apoptosis by Inducing Spindle Multipolarity via Centrosome Amplification and Declustering. Cell Death Differ 18 (4), 632–644. 10.1038/cdd.2010.133 21052096 PMC3131906

[B16] Kline-Smith S. L. Walczak C. E. (2004). Mitotic Spindle Assembly and Chromosome Segregation. Mol. Cel. 15 (3), 317–327. 10.1016/j.molcel.2004.07.012 15304213

[B17] Kollareddy M. Sherrard A. Park J. H. Szemes M. Gallacher K. Melegh Z. (2017). The Small Molecule Inhibitor YK-4-279 Disrupts Mitotic Progression of Neuroblastoma Cells, Overcomes Drug Resistance and Synergizes with Inhibitors of Mitosis. Cancer Lett. 403, 74–85. 10.1016/j.canlet.2017.05.027 28602975 PMC5542135

[B18] Lawo S. Bashkurov M. Mullin M. Ferreria M. G. Kittler R. Habermann B. (2009). HAUS, the 8-subunit Human Augmin Complex, Regulates Centrosome and Spindle Integrity. Curr. Biol. 19 (10), 816–826. 10.1016/j.cub.2009.04.033 19427217

[B19] Li M. Ke J. Wang Q. Qian H. Yang L. Zhang X. (2017). Upregulation of ROCK2 in Gastric Cancer Cell Promotes Tumor Cell Proliferation, Metastasis and Invasion. Clin. Exp. Med. 17 (4), 519–529. 10.1007/s10238-016-0444-z 27921230

[B20] Lodygin D. Menssen A. Hermeking H. (2002). Induction of the Cdk Inhibitor P21 by LY83583 Inhibits Tumor Cell Proliferation in a P53-independent Manner. J. Clin. Invest. 110 (11), 1717–1727. 10.1172/jci0216588 12464677 PMC151636

[B21] Meunier S. Vernos I. (2012). Microtubule Assembly during Mitosis - from Distinct Origins to Distinct Functions?. J. Cel Sci 125, 2805–2814. 10.1242/jcs.092429 22736044

[B22] Nakaoka Y. Miki T. Uehara R. Obuse C. Kubo M. Hiwatashi Y. (2012). An Inducible RNA Interference System in Physcomitrella Patens Reveals a Dominant Role of Augmin in Phragmoplast Microtubule Generation. Plant Cell 24 (4), 1478–1493. 10.1105/tpc.112.098509 22505727 PMC3398558

[B23] Nakayama G. Tanaka C. Kodera Y. (2013). Current Options for the Diagnosis, Staging and Therapeutic Management of Colorectal Cancer. Gastrointest. Tumors 1 (1), 25–32. 10.1159/000354995 26674429 PMC4645570

[B24] Paier C. R. K. Maranhão S. S. Carneiro T. R. Lima L. M. Rocha D. D. Santos R. D. S. (2018). Natural Products as New Antimitotic Compounds for Anticancer Drug Development. Clinics (Sao Paulo, Brazil) 73, e813s. 10.6061/clinics/2018/e813s PMC6256996 30540125

[B25] Po'uha S. T. Kavallaris M. (2015). Gamma-actin Is Involved in Regulating Centrosome Function and Mitotic Progression in Cancer Cells. Cell Cycle 14 (24), 3908–3919. 10.1080/15384101.2015.1120920 26697841 PMC4825712

[B26] Sánchez-Huertas C. Freixo F. Viais R. Lacasa C. Soriano E. Lüders J. (2016). Non-centrosomal Nucleation Mediated by Augmin Organizes Microtubules in post-mitotic Neurons and Controls Axonal Microtubule Polarity. Nat. Commun. 7, 12187. 10.1038/ncomms12187 27405868 PMC4947180

[B27] Shen A. Chen Y. Liu L. Huang Y. Chen H. Qi F. (2019). EBF1-Mediated Upregulation of Ribosome Assembly Factor PNO1 Contributes to Cancer Progression by Negatively Regulating the P53 Signaling Pathway. Cancer Res. 79 (9), 2257–2270. 10.1158/0008-5472.can-18-3238 30862720

[B28] Sherr C. J. (1996). Cancer Cell Cycles. Science 274 (5293), 1672–1677. 10.1126/science.274.5293.1672 8939849

[B29] Taylor B. F. McNeely S. C. Miller H. L. States J. C. (2008). Arsenite-induced Mitotic Death Involves Stress Response and Is Independent of Tubulin Polymerization. Toxicol. Appl. Pharmacol. 230 (2), 235–246. 10.1016/j.taap.2008.02.030 18485433 PMC2504415

[B30] Vescovo V. D. Denti M. A. (2015). microRNA and Lung Cancer. Adv. Exp. Med. Biol. 889, 153–177. 10.1007/978-3-319-23730-5_9 26659001

[B31] Vogelstein B. Lane D. Levine A. J. (2000). Surfing the P53 Network. Nature 408 (6810), 307–310. 10.1038/35042675 11099028

[B32] Wade Harper J. Adami G. R. Wei N. Keyomarsi K. Elledge S. J. (1993). The P21 Cdk-Interacting Protein Cip1 Is a Potent Inhibitor of G1 Cyclin-dependent Kinases. Cell 75 (4), 805–816. 10.1016/0092-8674(93)90499-g 8242751

[B33] Wang Q. He G. Hou M. Chen L. Chen S. Xu A. (2018). Cell Cycle Regulation by Alternative Polyadenylation of CCND1. Sci. Rep. 8 (1), 6824. 10.1038/s41598-018-25141-0 29717174 PMC5931507

[B34] Wang Z. Fan M. Candas D. Zhang T.-Q. Qin L. Eldridge A. (2014). Cyclin B1/Cdk1 Coordinates Mitochondrial Respiration for Cell-Cycle G2/M Progression. Dev. Cel. 29 (2), 217–232. 10.1016/j.devcel.2014.03.012 PMC4156313 24746669

[B35] Yuba-Kubo A. Kubo A. Hata M. Tsukita S. (2005). Gene Knockout Analysis of Two γ-tubulin Isoforms in Mice. Dev. Biol. 282 (2), 361–373. 10.1016/j.ydbio.2005.03.031 15893303

[B36] Zhu H. Coppinger J. A. Jang C.-Y. Yates J. R. Fang G. (2008). FAM29A Promotes Microtubule Amplification via Recruitment of the NEDD1-γ-Tubulin Complex to the Mitotic Spindle. J. Cel. Biol. 183 (5), 835–848. 10.1083/jcb.200807046 PMC2592839 19029337

