# Supplementary material for: Down-Regulating HAUS6 Suppresses Cell Proliferation by Activating the p53/p21 Pathway in Colorectal Cancer
Source: Front Cell Dev Biol. 2022 Jan 12;9:772077. doi: 10.3389/fcell.2021.772077 (PMC8790508; doi:10.3389/fcell.2021.772077)
Supplement: Supplementary file 1 [file DataSheet1.doc]

**Figure S1**

**
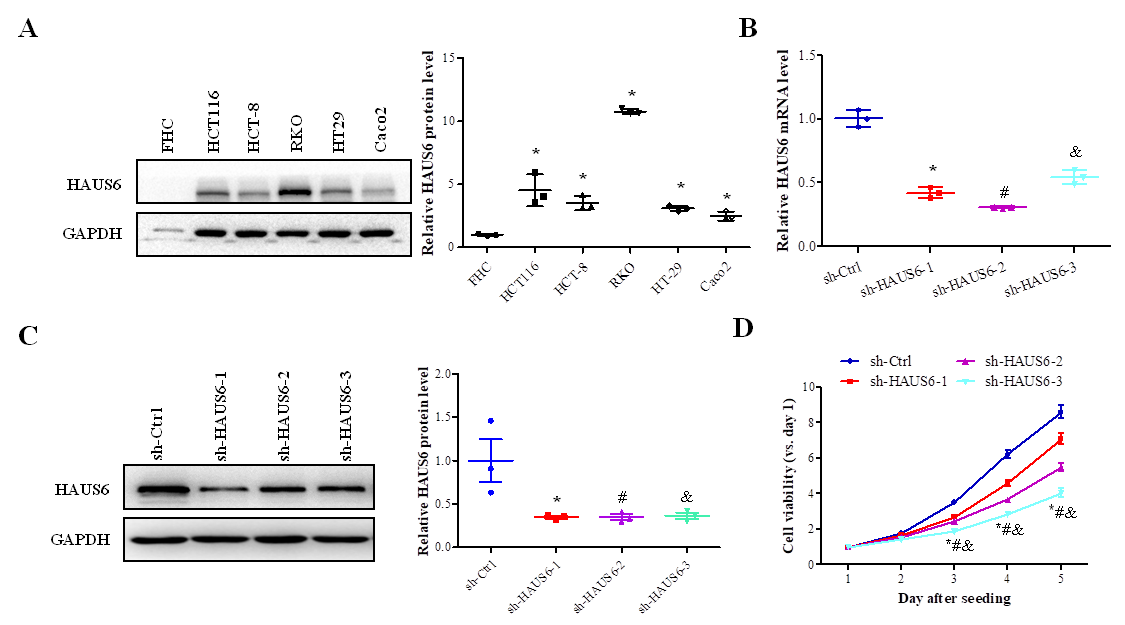
**

**Figure S1. shRNA-mediated knockdown of HAUS6 suppresses CRC cell growth.**

**(A)** Basal HAUS6 protein expression in a panel of CRC cell lines and human colon FHC cells were determined using western blot analysis. GAPDH was used as an internal control. Band intensities were quantified using ImageLab software. Mean±SD; n=3; **p*<0.05, vs. FHC, by independent Student's t test. **(B-C)** HCT116 cells were transfected with lentivirus encoding one of three anti-HAUS6 shRNAs or control shRNA (sh-Ctrl). Levels of HAUS6 **(B)** mRNA and **(C)** protein were determined using quantitative RT-PCR or western-bloting. GAPDH was used as an internal control. Band intensities were quantified using ImageLab software. Mean±SD; n=3; *#& *p*<0.05, vs. sh-Ctrl, by one-way ANOVA with LSD post hoc test. **(D)** Viability of HCT116 cells after transfected with lentivirus encoding one of three anti-HAUS6 shRNAs (sh-HAUS6) or control shRNA (sh-Ctrl) was determined by CCK-8 assay. Data are normalized to viability on day 1 and are represented as fold changes. Mean±SD; n=6; *#& *p*<0.05, vs. sh-Ctrl, by independent Student's t test.

**Figure S2**

**
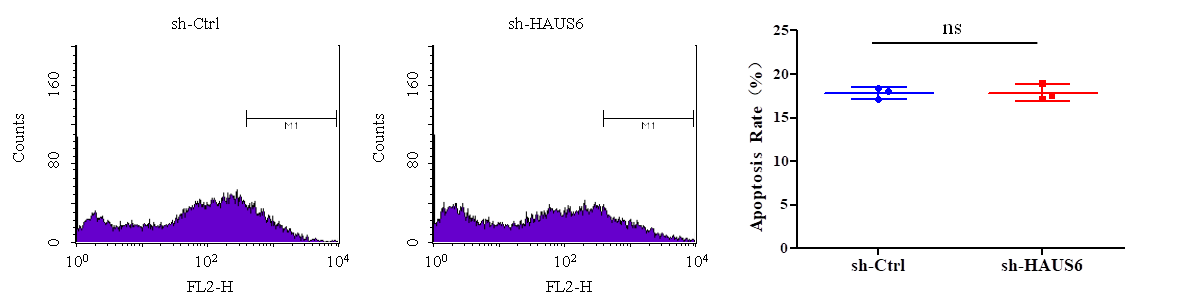
**

**Figure S2. The effect of HAUS6 knockdown on apoptosis of CRC cell.**

HCT116 cells were transfected with lentivirus encoding one of anti-HAUS6 shRNAs or control shRNA (sh-Ctrl). Apoptosis of HCT116 cells was determined by Annxin V staining followed by flow cytometry analysis. Mean±SD; n=3; compared by independent Student's *t* test.
