# Supplementary material for: Down-Regulating HAUS6 Suppresses Cell Proliferation by Activating the p53/p21 Pathway in Colorectal Cancer
Source: Front Cell Dev Biol. 2022 Jan 12;9:772077. doi: 10.3389/fcell.2021.772077 (PMC8790508; doi:10.3389/fcell.2021.772077)
Supplement: Supplementary file 2 [file DataSheet2.doc]

**Table S1: Oncomine Analysis of HAUS6 Expression in Colorectal Cancer (Total 7 Colorectal Cancer Cohorts)**

| Cohort no. | Cohort | Data type | Sample (n) | Fold-change | P value |
| --- | --- | --- | --- | --- | --- |
| 1 | Sabates-Bellver Colon | cDNA microarray | Rectal adenoma (7) vs normal (32) | 3.593 | 1.38E-12 |
|  |  |  | Colon adenoma (25) vs normal (32) | 2.296 | 1.56E-12 |
| 2 | TCGA | RNAseq | Cecum adenocarcinoma (22) vs normal (22) | 3.241 | 4.28E-17 |
|  |  |  | Colon Mucinous Adenocarcinoma (22)vs normal (22) | 2.891 | 9.39E-16 |
|  |  |  | Rectal adenocarcinoma (60) vs normal (22) | 2.517 | 4.36E-17 |
|  |  |  | Colon adenocarcinoma (101) vs normal (22) | 2.889 | 1.13E-19 |
|  |  |  | Rectal Mucinous Adenocarcinoma (6) vs normal (22) | 2.592 | 3.00E-03 |
| 3 | Kaiser Colon | cDNA microarray | Rectosigmoid adenocarcinoma (10) vs normal (5) | 2.396 | 1.32E-06 |
|  |  |  | Cecum adenocarcinoma (17) vs normal (5) | 2.186 | 6.22E-17 |
|  |  |  | Rectal adenocarcinoma (8) vs normal (5) | 2.29 | 6.47E-05 |
|  |  |  | Colon mucinous adenocarcinoma (13) vs normal (5) | 2.181 | 2.13E-06 |
|  |  |  | Colon adenocarcinoma (41) vs normal (5) | 2.199 | 4.00E-07 |
|  |  |  | Rectal mucinous adenocarcinoma (4) vs normal (5) | 2.061 | 7.29E-04 |
| 4 | Skrzypczak Colorectal 2 | cDNA microarray | Colon Adenoma (5)vs normal (10) | 3.629 | 5.15E-07 |
|  |  |  | Colon carcinoma (5) vs normal (10) | 2.973 | 1.81E-08 |
| 5 | Hong Colorectal | cDNA microarray | Colorectal carcinoma (70) vs normal (10) | 3.097 | 3.04E-09 |
| 6 | Gaedcke Colorectal | cDNA microarray | Rectal adenocarcinoma (65) vs normal (65) | 1.936 | 1.54E-28 |
| 7 | Skrzypczak Colorectal | cDNA microarray | Colorectal carcinoma (36) vs normal (24) | 1.856 | 5.86E-10 |
|  |  |  | Colorectal adenocarcinoma (45) vs normal (25) | 1.783 | 6.80E-10 |

**Table S2: Clinicopathologic characteristics of patients**

| Characteristic | n (%) |
| --- | --- |
| Age (years) |  |
| < 65 | 24 (67) |
| ≥ 65 | 12 (33) |
| Gender |  |
| Male | 21 (58) |
| Female | 15 (42) |
| Tumor location |  |
| Colon | 21 (58) |
| Rectum | 15 (42) |
| Clinical stage |  |
| Ⅰ | 5 (14) |
| Ⅱ | 15 (42) |
| Ⅲ | 13 (36) |
| Ⅳ | 3(8) |
| Lymph node metastasis | 10 (28) |
| Distant metastasis | 3 (8) |

**Table S3: Clinicopathologic characteristics of patients**

| Characteristic | n (%) |
| --- | --- |
| Age (years) |  |
| < 65 | 37 (46) |
| ≥ 65 | 43 (54) |
| Gender |  |
| Male | 47 (59) |
| Female | 33 (41) |
| Clinical stage |  |
| Ⅰ | 8 (1) |
| Ⅱ | 37 (46) |
| Ⅲ | 33 (41) |
| Ⅳ | 2 (2) |
| Lymph node metastasis | 116 (41) |
| Distant metastasis | 9 (3) |

**Table S4: Clinicopathologic characteristics of patients**

| Characteristic | n (%) |
| --- | --- |
| Age (years) |  |
| < 65 | 116 (41) |
| ≥ 65 | 164 (59) |
| Gender |  |
| Male | 161 (58) |
| Female | 119 (42) |
| Tumor size |  |
| < 5 cm | 113 (40) |
| ≥ 5 cm | 167 (60) |
| Clinical stage |  |
| Ⅰ | 33 (12) |
| Ⅱ | 127 (45) |
| Ⅲ | 111 (40) |
| Ⅳ | 9 (3) |
| Lymph node metastasis | 116 (41) |
| Distant metastasis | 9 (3) |

**Table S5: Primer sequences for quantitative RT-PCR**

| Target gene | Primers (5`-3`) |
| --- | --- |
| GAPDH | F: ATGGGGAAGGTGAAGGTCG  R: GGGGTCATTGATGGCAACAATA |
| CDKN1A (p21) | F: TAGCAGCGGAACAAGGAG  R: AAACGGGAACCAGGACAC |
| HAUS6 | F: CTTTCGAGAAGGAGCATCTCTG  R: GATCTTTCCGCAGGCAATGGT |
| TP53 | F: TAACAGTTCCTGCATGGGCGGC R: AGGACAGGCACAAACACGCACC |

F, Forward; R, Reverse

**Table S6: The antibodies for western blot or Immunohistochemistry**

| **Antigen or description** | **Application(dilution)** | **Origin** |
| --- | --- | --- |
| HAUS6 antibody | Western-blot (1:1000), IHC (1:500) | GTX118732, Genetex, USA |
| p53 antibody | Western-blot (1:1000) | 10442-1-AP, Proteintech, USA |
| p21 antibody | Western-blot (1:1000) | 2947S, CST, USA |
| GAPDH polyclonal antibody | Western-blot (1:1000) | Abp57259, Abbkine, China |
| KI-67 antibody | IHC (1:500) | ab15580, abcam, USA |
| Anti-rabbit IgG, HRP-linked Antibody | Western-blot (1:5000) | #7074, CST, USA |

HRP, horseradish peroxidase; IHC, immunohistochemistry

**Table S7: The double-stranded shRNAs targeting each gene**

| Target gene | Code | Sequence* |
| --- | --- | --- |
| HAUS6 | sh-HAUS6-1 | CAGAATCATTACCTGTGTT |
| HAUS6 | sh-HAUS6-2 | ACATAAGCAACATAACCAA |
| HAUS6 | sh-HAUS6-3 | GTCAAGAATGCCTCTCTTA |
| CDKN1A | sh-CDKN1A | CTCTACATCTTCTGCCTTA |
| Control | sh-Ctrl | TTCTCCGAACGTGTCACGT |

All oligonucleotides were double-stranded. In the case of shRNAs, only the 5'-3' strand is shown.
